# Imaging of PDE2- and PDE3-Mediated cGMP-to-cAMP Cross-Talk in Cardiomyocytes

**DOI:** 10.3390/jcdd5010004

**Published:** 2018-01-19

**Authors:** Nikoleta Pavlaki, Viacheslav O. Nikolaev

**Affiliations:** 1 Institute of Experimental Cardiovascular Research, University Medical Center Hamburg-Eppendorf, 20246 Hamburg, Germany; n.pavlaki@uke.de; 2 German Center for Cardiovascular Research (DZHK), Partner Site Hamburg/Kiel/Lübeck, 20246 Hamburg, Germany

**Keywords:** cAMP, cGMP, phosphodiesterase, FRET, imaging, cross-talk

## Abstract

Cyclic nucleotides 3′,5′-cyclic adenosine monophosphate (cAMP) and 3′,5′-cyclic guanosine monophosphate (cGMP) are important second messengers that regulate cardiovascular function and disease by acting in discrete subcellular microdomains. Signaling compartmentation at these locations is often regulated by phosphodiesterases (PDEs). Some PDEs are also involved in the cross-talk between the two second messengers. The purpose of this review is to summarize and highlight recent findings about the role of PDE2 and PDE3 in cardiomyocyte cyclic nucleotide compartmentation and visualization of this process using live cell imaging techniques.

## 1. Introduction

Cyclic nucleotides 3′,5′-cyclic adenosine monophosphate (cAMP) and 3′,5′-cyclic guanosine monophosphate (cGMP) are ubiquitous intracellular second messengers that regulate multiple physiological functions as well as pathological conditions. In cardiomyocytes, there are at least three pathways that normally trigger their production after initial first messenger stimuli: (i) the β-adrenergic pathway for cAMP production, (ii) the nitric oxide, and (iii) the natriuretic peptide (NP) receptor pathways for cGMP synthesis.

### 1.1. The cAMP and β-Adrenergic Pathway

In healthy cardiomyocytes, sympathetic activation mainly via β-adrenergic receptor (β-AR) signaling leads to the production of cAMP and thereby to increased contractile force (inotropy), heart rate (chronotropy), and cell relaxation (lusitropy) [1]. When a ligand binds to a G protein-coupled receptor (GPCR) located on the plasma membrane, a conformational change occurring in the receptor leads to G-protein activation. Activated G-proteins can in turn, activate or inhibit cAMP-forming enzymes adenylyl cyclases (ACs) which generate cAMP from ATP. Subsequently, cAMP acts in cells via one or more of the following effector proteins:

(a) cAMP-dependent protein kinase (PKA), which is responsible for phosphorylation of several calcium handling proteins involved in cardiac excitation-contraction coupling (ECC) including L-type Ca^2+^ channel (LTCC) at the plasmalemma, phospholamban, and ryanodine receptors at the sarcoplasmic reticulum (SR), myosin-binding protein C, and troponin I at the myofilaments [1,2]. PKA is the main effector protein in the cAMP cascade, while Ca^2+^-inhibited AC5 and AC6 are the predominant cAMP generating adenylyl cyclases in adult (AC5 and AC6) and fetal (AC6) ventricular cardiac tissue [3];
(b) exchange proteins directly activated by cAMP (Epac1 and Epac2) [4], which are implicated in pathological cardiomyocyte growth [5,6];
(c) cyclic nucleotide gated ion channels (CNGCs) including HCN channels located in the sinus node, which regulate the capacity of cardiac cells to initiate spontaneous action potentials (automaticity) [7,8,9];
(d) the recently introduced Popeye-domain-containing proteins which affect cardiac pacemaking [10,11].

### 1.2. NO/sGC/cGMP Pathway

Biosynthesis of cGMP is catalyzed by two discrete guanylyl cyclase (GC) families, one being activated in the presence of nitric oxide (NO) and called soluble guanylyl cyclase (sGC) and the other acting as membrane receptors for natriuretic peptides (NPs), also called particulate guanylyl cyclase (pGC).

NO, alternatively known as “endothelial-derived relaxant factor” (EDRF) [12,13], is produced for example by endothelial cells after acetylcholine administration. It increases cGMP levels, activates cGMP-dependent protein kinase (PKG), and behaves in a way similar to nitrovasodilators [14,15]. Seminal work on the field [16,17,18] has firmly established that NO is produced by a family of NO biosynthetic enzymes called nitric oxide synthases (NOS). It includes neuronal (NOS-1 or nNOS), inducible (NOS-2 or iNOS), and endothelial nitric oxide synthases (NOS-3 or eNOS) [19], all of which having been detected in heart and vessels [20,21,22,23,24]. iNOS is an inducible biosynthetic enzyme, while eNOS and nNOS are both constitutive and inducible enzymes [25]. NO activates sGC by binding to both heme and non-heme sites [26,27,28], which leads to the production of cGMP [29] and its subsequent downstream effects [25,30,31].

### 1.3. NP/pGC/cGMP Pathway

Natriuretic peptides (NPs) constitute important cardiovascular regulators of inotropy and blood pressure [32] with atrial (ANP), brain (BNP), and C-type natriuretic peptides (CNP) being the most well-known ligands. In response to neurohumoral (catecholamines or angiotensin II) or mechanical (e.g., increased myocardial stretch or blood pressure) stimuli [33,34], ANP and BNP are produced and released by the atria and the ventricles of the heart, while CNP is produced mainly by endothelial cells of the vasculature [34].

These NPs can bind and activate several pGCs, two of which are expressed in the heart and exert the majority of their physiological effects. NPR1 (also called NPR-A or GC-A) is the receptor that binds both ANP and BNP with relatively high affinity (ANP > BNP) [35,36,37]. After ligand binding at its extracellular domain, pGCs undergo a conformational change upon which its intracellular domain generates cGMP [25]. As a widely distributed receptor in the cardiovascular system (heart, vessels, and kidneys), NPR1 regulates blood pressure, exerts antihypertrophic action, and preserves body homeostasis [35,36,37]. NPR2 (also called NPR-B or GC-B) is the CNP-specific receptor responsible for vascular regeneration and endochondral ossification. It is mainly localized in fibroblasts [38], the sympathetic nervous system [39], and the vascular endothelium and smooth muscle [40] and exerts antihypertrophic effects in cardiomyocytes [41,42].

Both NO/sGC and NP/pGC pathways stimulate cGMP synthesis and participate in the homeostasis of the cardiovascular system via (i) PKG-mediated protein phosphorylation [29,30,31], (ii) the activation of CNGCs, and (iii) the regulation of PDEs [25]. Physiologically, cGMP binds to specific sites in the regulatory domains of PKG, CNGC, or PDE in order to induce conformational changes and downstream effects. Disruption of downstream cascade at any level can initiate pathophysiological effects and may lead to hypertension, atherosclerosis, pulmonary hypertension, hypertrophy, ventricular remodeling, myocardial ischemia, dystrophy-related cardiomyopathies, mitochondrial metabolism, or heart failure [25].

Apart from the classical cyclic nucleotides, cyclic cytidine (cCMP) and cyclic uridine monophosphates (cUMP) have been recently introduced as non-canonical second messengers generated by ACs and GCs [43,44]. However, available published data provides limited information regarding their effector proteins and physiological significance, so further studies are required to fully elucidate their role in the cardiovascular system.

## 2. Compartmentation of cAMP and cGMP Signaling

The fact that multiple receptor stimuli can trigger diverse intracellular effects generated via the production of just a few second messengers such as cAMP and cGMP led to a currently accepted theory of cyclic nucleotide compartmentation. Compartmentation refers to the mechanisms by which multiple spatially segregated cAMP/PKA and cGMP/PKG signaling pathways exert different or even opposing functional effects in distinct subcellular microdomains of the same cell [9,45]. It appears to be of critical importance for cardiovascular system, since local cyclic nucleotide actions and the interplay of the cAMP and cGMP signaling pathways have been implicated in physiological functions or pathological conditions.

Several proteins [46,47,48,49,50] contribute to cyclic nucleotide compartmentation, which spatially, temporally, and functionally controls the downstream effects of cyclic nucleotides (extensively studied for cAMP) in the cardiovascular system [25,51,52,53]. They include (a) GPCRs located in lipid rafts [54,55], at transverse tubules [56] and in non-caveolar membrane domains [57]; (b) ACs and GCs [58,59]; (c) Scaffold proteins [60,61,62]) such as A-kinase anchoring proteins (AKAPs) [52,63,64] and Calveolin-3 [54,65,66,67]; (d) physical barriers—e.g., mitochondria, cAMP buffering by PKA, cAMP export [68,69] are some of the mechanisms that create locally confined intracellular domains regulating signaling; and (e), the most prominent and extensively studied of all, the PDE-mediated hydrolysis of cyclic nucleotides, which is of high pharmacological and clinical interest [64,70,71].

PDEs can control cAMP and cGMP compartmentation by providing their local hydrolytic degradation and creating spatial second messenger gradients [72]. Although much fewer scientific data are available on cGMP compartmentation, the role of PDEs in local confinement of cGMP pools has recently been elucidated, especially that of PDE2, PDE5, and PDE9 [25,73]. Furthermore, spatial organization of PKG and GCs in distinct subcellular complexes appears to be another important aspect of cGMP microdomain regulation [74]. It still remains to be established whether, for example, myosin, NPR1, and troponin T could act as PKG scaffolding proteins [75].

Among the relevant experimental evidence, studies on knockout mice do also highlight the importance of the crucial role PDEs play in the cAMP/cGMP signaling pathways and their respective crosstalk [76]. The interplay among the β-adrenergic and NO/cGMP/PKG pathways can be interpreted as a network phenomenon arising from the molecular selectivity of PDEs to cAMP and cGMP [77].

## 3. Phosphodiesterases (PDEs)

**Figure 1:**
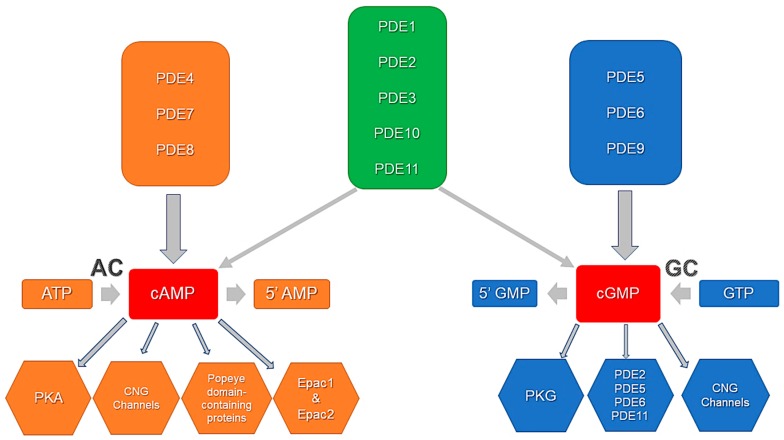
PDE activity, specificity, and cyclic nucleotide effector proteins. PDE4, PDE7, and PDE8 selectively degrade cAMP. PDE5, PDE6, and PDE9 selectively degrade cGMP. PDE1, PDE2, PDE3, PDE10, and PDE11 are dual-specificity phosphodiesterases that hydrolyze both cAMP and cGMP. Downstream effectors include PKA, PKG, Epacs, PDEs, CNG channels and Popeye domain-containing proteins. Adapted from Ahmad et al., 2015 [76].

PDEs are the hydrolyzing enzymes that terminate intracellular effects of cyclic nucleotides by their hydrolysis to fine-tune the signaling and to prevent continuous activation of the downstream effector proteins. These cyclic nucleotide-degrading enzymes constitute one of the most important mechanisms, by which cyclic nucleotides are spatially, temporally, and functionally compartmentalized in cardiomyocytes and other cells. Of the 12 PDE families [78,79], there are seven, namely PDE1 [80], PDE2 [81], PDE3 [76], PDE4 [82], PDE5 [83], PDE8 [84], and PDE9 [73] that have been reported to be expressed and active in mammalian cardiomyocytes (Figure 1). They are an integral part of the multimolecular signaling/regulatory complexes, i.e., signalosomes [52,64,76,84]. This review will particularly explore the so-called cGMP-regulated PDEs, especially PDE2 and PDE3, which critically regulate cGMP-to-cAMP cross-talk and cyclic nucleotide actions in cardiomyocyte microdomains.

### 3.1. Phosphodiesterase 2

**Figure 2:**
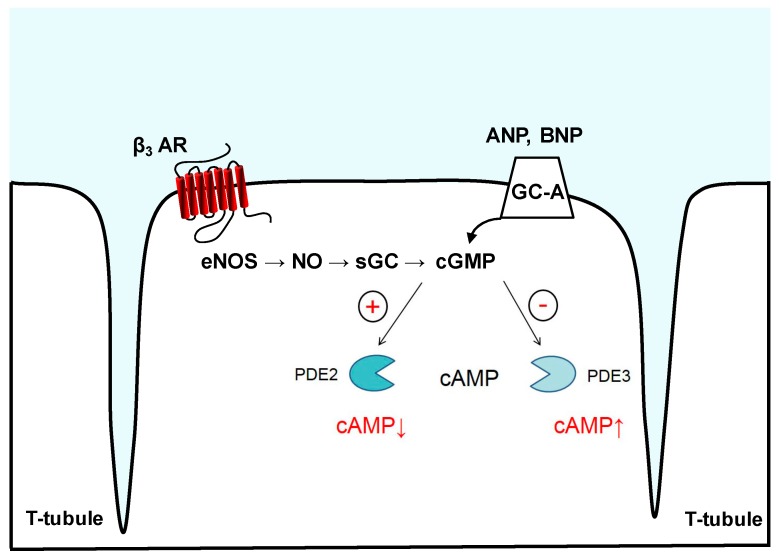
PDE2- and PDE3-mediated cyclic nucleotide crosstalk. cGMP synthesis occurs by pGCs such as GC-A, which serves as a membrane receptor for ANP and BNP, or by NO-activated sGC, e.g., downstream of eNOS and β_3_-adrenoreceptor (β_3_-AR). Binding of cGMP to PDE2 can allosterically increase its hydrolytic activity, lowering cAMP levels in subcellular microdomains. PDE3 is a “cGMP-inhibited” phosphodiesterase that upon cGMP binding and degradation in the catalytic domain shows reduced rates of cAMP hydrolysis, generating a positive cGMP-to-cAMP cross-talk.

PDE2 is a dual-substrate enzyme, which hydrolyzes both cAMP and cGMP with similar maximal rates in bovine adrenal and heart tissues [85]. Only one gene (*Pde2a*) gives rise to three known PDE2A isoforms, which are differentially located in the cytosol, mitochondria, and cellular membranes [58]. It is characteristic of this PDE family that the cGMP-mediated control of cAMP hydrolysis arises, when cGMP binds allosterically to the GAF-B domain of PDE2A, so that cAMP hydrolysis occurs with a 10-fold higher rate [86,87,88]. In this manner, cGMP via PDE2A is able to negatively regulate cAMP levels [51] and therefore to initiate a negative cGMP-to-cAMP cross-talk [89] (Figure 2). Initially cloned from rat brain [90] and purified from bovine or calf tissues (heart, liver adrenal gland, and platelets) [85,91], the PDE2A protein is also found in endothelial cells, macrophages, and brain [92,93]. Platelet aggregation [94], aldosterone secretion [95], and regulation of calcium channels [96] require PDE2A-mediated hydrolysis of cAMP. Recently, a PDE2A isoform regulating the mitochondrial respiratory chain has been detected, discovering a possible new pathway for the drug-induced control of mitochondrial function [97]. Of particular importance are those studies referring to PDE2A expression in isolated cardiomyocytes and myocardium. In cardiomyocytes, PDE2A together with PDE5 is also involved in the degradation of sGC-synthesized cGMP, whereas pGC-synthesized cGMP is preferentially hydrolyzed by PDE2A [72,98,99].

### 3.2. Phosphodiesterase 3

Another important cGMP-regulated PDE is PDE3. This enzyme hydrolyzes both cAMP and cGMP. Often referred to as cGMP-inhibited PDE, PDE3 shows higher catalytic rates for cAMP but relatively high affinity for cGMP, which acts as a competitive inhibitor of cAMP hydrolysis [100,101]. This creates the so-called positive cGMP-to-cAMP cross-talk (Figure 2). PDE3A and PDE3B are the two PDE3 subfamilies, with the former being abundant in cardiomyocytes, oocytes, vascular smooth muscle and platelets and the latter being expressed in the pancreas, liver, and adipose tissue [100]. PDE3A controls myocardial contractility by interacting with the sarcoplasmic/endoplasmic reticulum calcium ATPase (SERCA2a) [102]. By utilizing their direct positive inotropic effects, PDE3 inhibitors are used for acute treatment of end-stage heart failure (HF) [103], albeit presenting increased mortality as well as incidence of arrhythmias and sudden death after chronic use [104,105]. In contrast, PDE3B seems to be more actively engaged in energy metabolism [106,107], but it can also protect the heart from ischemia/reperfusion injury [108]. Knockout models have revealed that PDE3A, but not PDE3B, exerts inotropic and chronotropic effects after treatment with PDE3 inhibitors [109] because PDE3A regulates SERCA2a activity and subsequent SR Ca^2+^ uptake [102]. By chronically suppressing its expression or action, myocyte apoptosis in vitro [110] or deterioration of ischemia/reperfusion-induced apoptosis and cardiac injury in vivo [111] have been observed. Similarly, disruption of PDE3B interaction with phosphoinositide 3-kinase γ, which can serve as an AKAP, has deleterious effects [112,113,114] such as arrhythmias [114], necrotic cardiac tissue damage, and fibrosis [112].

PDE3 along with other PDEs constitutes an integral part of cAMP degradation. Evidence suggests that it may also controls cGMP levels [108,115], atrial dynamics, and myocyte ANP release, depending on the involved induction mechanism [72,116]. In terms of cGMP and cAMP pathway interactions, cGMP binding to PDE2 enhances the hydrolytic activity of the enzyme and enables the negative cGMP-to-cAMP cross-talk [75]. Conversely, cGMP binding to the catalytic domains of PDE3 reduced the rate of cAMP degradation, thereby mediating the positive cGMP-to-cAMP cross-talk. The previously reported experimental data regarding the affinity, specificity, and enzymatic activity of PDE2 and PDE3 can largely explain their crucial role in cGMP/cAMP crosstalk [75].

## 4. Visualization of Compartmentalized cAMP and cGMP

Initially, the idea of compartmentalized action of cyclic nucleotides was conceived [117] and revealed by several research groups [118,119,120,121,122] with Buxton and Brunton (1983) [122] using classical biochemical methods to show that prostaglandin induces different PKA activity rates in particulate and soluble fractions of cardiac myocytes after cAMP generation. Later on, Juvericius and Fischmeister (1996) [123], by utilizing a combination of two-barrel microperfusion and whole patch clamp techniques, further confirmed the compartmentation theory in frog ventricular cells, where local application of a β-adrenergic agonist preferentially stimulated the LTCCs close to activated receptors.

To detect cAMP compartmentation in health and disease, multiple techniques have been employed that were only able to detect global concentrations of cyclic nucleotides and required plenty of tissue material [124]. However, biochemical (radio- and enzyme-linked immunoassays) or even electrophysiological approaches (patch-clamp technique), though sensitive and specific, are limited in their capability to record and analyze cyclic nucleotide gradients directly in subcellular microdomains under physiological conditions [124]. Therefore, novel live cell imaging techniques have been developed for the visualization of cyclic nucleotide signaling and its compartmentation in real time with high temporal and spatial resolution [9,124]. Such techniques are mostly based on Förster Resonance Energy Transfer (FRET) biosensors.

FRET biosensors report a non-radiative energy transfer from an excited fluorescent molecule that acts as a donor to a neighboring (located at nm distance) molecule that acts as an acceptor with subsequent fluorescence emission without the direct excitation of the acceptor [125]. Multiple FRET-based biosensors for cGMP [115,126,127,128,129,130] and cAMP [81], and for the activity of the downstream effector proteins such as PKA [131,132,133,134,135,136], Epac [137,138,139,140,141], or CNG channels [142,143,144], have been developed and successfully used to visualize cGMP and cAMP gradients [124,145,146]. They can be further combined with other techniques such as scanning ion conductance microscopy (SICM), which can be used to deliver receptor ligands onto defined membrane structures to targeted distinct cAMP or cGMP pools and to study receptor–microdomain interactions. SICM is a non-optical imaging technique that uses a small glass nanopipette to obtain a highly resolved morphological profile of a living cell membrane based on ion current measurement [147,148,149,150]. It can also be combined with FRET for more accurate and specific detection of microdomain alterations in health and disease [149,151,152].

## 5. Imaging of cGMP-to-cAMP Crosstalk via PDE2 and PDE3

Employing FRET for live cell imaging, recent studies have revealed strongly remodeled cAMP/cGMP microdomains and subcellular concentration profiles in various cardiac pathologies, leading among other mechanisms to a putatively enhanced involvement of PDE2 in cAMP/cGMP breakdown and crosstalk compared to the other cardiac PDEs.

As mentioned above, the hydrolytic activity of PDE2 can be allosterically stimulated by cGMP to limit cAMP levels, referred to as a negative cGMP-to-cAMP crosstalk. In cardiomyocytes, cGMP can be produced by either pGC after ANP, BNP, and CNP stimulation or by the NO-dependent sGC. Sources for NO include both synthesis in other cell types (e.g., by endothelial cells) and inside cardiomyocytes, e.g., by β_3_-adrenoreceptor (β_3_-AR) stimulated pathway, which via inhibitory G-proteins leads to NOS activation (Figure 2). PDE2 hydrolyzes cAMP (e.g., produced in response to the β_1/2_-adrenergic agonists such as noradrenaline), but its stimulation can be in turn limited by its cGMP hydrolyzing activity, which increases in importance when cGMP concentration rises [51,89]. It has been suggested that PDE2-dependent cAMP hydrolysis might have a more critical effect on cardiomyocyte function, at least under adrenergic overdrive conditions [81,153,154].

The specific role of PDE2 in orchestrating the cyclic nucleotide compartmentation (i.e., cAMP) was supported by experimental evidence coming from a study that demonstrated that, in neonatal rat ventricular myocytes, activation of PDE2 was ineffective in counteracting the forskolin-mediated rise in intracellular cAMP levels [81]. It could also be inferred that, at least in part, stimulation of PDE2-mediated cAMP hydrolysis occurs via a β_3_-AR/eNOS/sGC pathway (Figure 2). On the contrary, evidence from other studies [155,156,157,158] showed that PDE2 was effective in blocking intracellular increases of cAMP levels mediated by catecholaminergic activation of β-adrenergic receptors or forskolin-mediated AC activation under hypertrophic conditions. By inhibiting the subsequent inotropic effects, these groups were able to argue for a distinct subcellular localization and activity of PDE2 within cardiomyocytes.

More recently, Mehel and colleagues [155] were able to show that myocardial PDE2 is unregulated in human and experimental heart failure and blocks cAMP increase after acute β-AR stimulation. PDE2 upregulation may act as a counterbalance, neutralizing neurohormonal (i.e., β-adrenergic) hyperactivity typically seen in heart failure [155,159]. Furthermore, specific PDE2 inhibition has restored β-AR-mediated signal in diseased cardiomyocytes, while PDE2 overexpression has completely abolished catecholamine effects and hypertrophy without affecting basal contractility [155]. In addition, cAMP hydrolysis via PDE2 mediated the reduction of aldosterone production in adrenal cells, suggesting beneficial synergy between cardiovascular and renal systems [88]. However, for every experimental study, the limitations dictated by the in vitro acquired results might not reflect the in vivo PDE functions, and further experiments in large animal models are required to fully explore the PDE2 role in heart failure pathophysiology. Nevertheless, the overexpressed PDE2 activity may constitute a potential approach to effectively control the deleterious effects of heart failure, e.g., by augmenting its microdomain-specific actions.

On the other hand, there are also studies in which PDE2 may not necessarily exert beneficial effects, but rather contribute to hypertrophy. In cell-based experiments, another pool of cAMP/PDE2 was found to modulate hypertrophic growth of cardiac myocytes by regulating PKA-dependent phosphorylation of nuclear factors of activated T cells (NFAT) [156]. In this study, Zoccarato and colleagues [156] showed that PDE3 and PDE4 inhibition increase cAMP levels and result in hypertrophy, whereas PDE2 inhibition is antihypertrophic despite an increase in cellular cAMP content. Live cell imaging of intact cardiomyocytes revealed that PDE2 inhibition exerted its antihypertrophic effects by generating a locally confined cAMP microdomain, in which PKA type II plays a significant role by phosphorylating NFAT. These are clearly contradicting reports showing remarkable discrepancies especially in the in vivo actions of cardiac PDE2. Further experimental work is required to fully elucidate this question as well as the role of PDE2 in different subcellular cAMP microdomains. It will be especially important to develop and study a tissue-specific knockout mouse model for PDE2.

Another live cell imaging study has developed the first in vivo model expressing a cAMP biosensor targeted to SERCA2a in transgenic mouse cardiomyocytes [157]. Using FRET imaging, it was able to unveil impaired cAMP signal communication between β_1_-AR located at the membrane and sarcoplasmic reticulum microdomains during early heart failure. By inhibiting PDE2, the authors demonstrated its higher contribution to the regulation of local cAMP levels under pathological conditions [157]. These data suggest that PDE2, when locally or globally upregulated, might potentially contribute to cardioprotective effects in certain microdomains.

**Figure 3:**
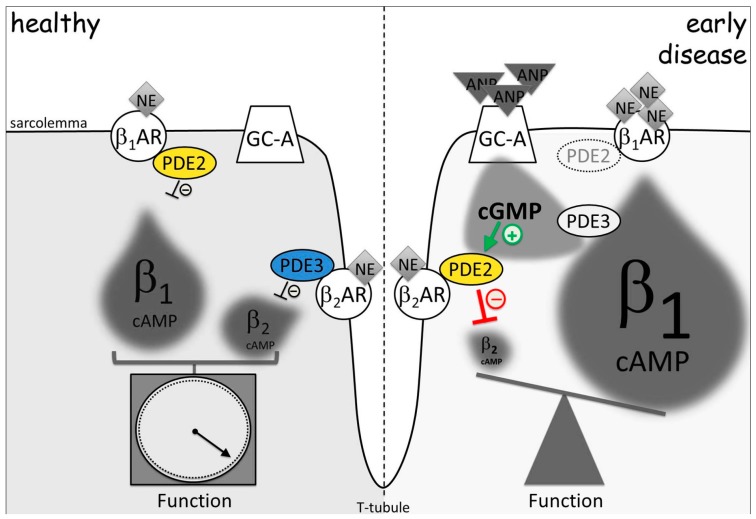
PDE2 and PDE3 redistribution in early cardiac hypertrophy changes cGMP-to-cAMP cross-talk. β_2_-AR microdomain is normally controlled by PDE3, while PDE2 is functionally associated with β_1_-AR. In disease, redistribution of PDE2 from β_1_- to β_2_-AR-associated membrane microdomains leads to a decrease of the local β_2_-AR-cAMP and to an increase of global β_1_-AR-cAMP pool under elevated ANP and cGMP levels observed in hypertrophy. This relocation of cGMP-regulated PDEs leads to a turnaround of cGMP-to-cAMP cross-talk between both β-AR microdomains. By this mechanism, elevated ANP can augment β-adrenoceptor-stimulated contractile function. NE: norepinephrine, the physiological β-AR agonist. Adapted from [158].

Moreover, an elegantly-designed study by Perera et al. [158] proved experimentally for the first time that, in early compensated cardiac hypertrophy preceding heart failure, cGMP-sensitive PDE2 and PDE3 were already physically and functionally rearranged between β_1_- and β_2_-AR-associated cAMP microdomains despite unchanged whole cell expression levels and activities. More specifically, the switch of PDEs from PDE3 to PDE2 at the β_2_-AR, accompanied by a reduction of PDE2 at the β_1_-AR, led to a turnaround of cAMP cross-talk in a way that, in this pathological setting, the ANP/cGMP signaling pathway by this mechanism could enhance β-AR-mediated cardiac contractility inducing positive inotropic and chronotropic effects following β-AR stimulation (Figure 3). The provided evidence shed light on the poorly understood early microdomain remodeling mechanisms. It has been suggested that, in this way, the heart can compensate for the increased contractility demand under pressure overload [158]. However, our knowledge about microdomain-related contractility mechanisms in early disease is still in its infancy and has to be improved.

The ability of PDE2 to compartmentalize local pools of cAMP has been in part attributed to a much higher speed of cAMP hydrolysis by this PDE as compared to its synthesis by ACs based on FRET imaging in aldosterone producing cells [139]. The most recent finding in regard to cGMP-sensitive cyclic nucleotide compartmentation via PDE2/3 is described in a study using a cardiomyocyte-specific PDE2 transgenic mouse model [159]. In fact, it was shown that endogenous PDE2 contributes to heart rate control under physiological conditions and that PDE2 overexpression protects against arrhythmias and enhances inotropic performance after myocardial infarction [159], providing evidence in support of PDE2 overexpression and highlighting its beneficial role in diseased heart. However, the conclusions from such data obtained from a transgenic mouse model overexpressing this PDE several folds above the endogenous level should be treated with caution since excessive amounts of PDE2 might vanish the boundaries between at least some cAMP microdomains.

Trying to further elucidate the NO/cGMP or NPs/cGMP and cAMP crosstalk, which was also demonstrated in CMs, additional studies utilizing previously developed targeted FRET biosensors [160] and live cell imaging techniques uncovered that the interconnection between cGMP and cAMP in CMs is closely linked to the intracellular locus of regulation [161]. Depending on the recruited cyclase (soluble or particulate) and the associated PDE, cGMP can either augment or inhibit the cAMP levels after catecholamine stimulation and further affect downstream phosphorylation of PKA and contractility. In fact, cGMP can inhibit PDE3 as a competitive substrate for cAMP and allosterically stimulate PDE2A-mediated cAMP hydrolysis [72] locally without largely affecting global cAMP levels in the cell [161]. Induction of cGMP by catecholamine stimulation was found to differentially regulate intracellular cAMP pools that either activate PKA-RI/PDE3- or PKA-RII/PDE2-associated compartments and provoke opposing effects on local cAMP signals [161]. Low basal cGMP levels (~10–50 nmol/L) which could be detected by FRET in adult cardiomyocytes [115] can even facilitate cGMP hydrolyzing activity of PDE3, while higher (between 200 and 500 nmol/L) cGMP levels can activate PDE2A and inhibit PDE3 towards cAMP hydrolysis [162]. Similarly, NO donors via sGC affect both the PKA-RI and PKA-RII compartments, whereas ANP via pGC limits cGMP action to the PKA-RII compartment only [161]. This evidence supports that cGMP exerts local but not global cAMP control in the cardiomyocyte in neonatal rat ventricular myocytes (NRVMs) when isoproterenol is administered in a microdomain-specific manner [161]. For instance, cGMP diminishes cAMP gradients by PDE2 activity when sGC and ANP/pGC mediate its production, while it augments cAMP gradients by inhibiting PDE3 when sGC does so. It is evident that PDE2 exerts cardioprotective regulation against excessive adrenergic stimulation by interconnecting β_1/2-_AR/cAMP and β_3_-AR/cGMP pathways [163] and paves the way for further experimental exploration.

Recently, Li and colleagues (2015) [164] showed that PDE2A overexpression blunted BNP-mediated effects by decreasing cGMP production and negatively affecting downstream effectors such as calcium current, intracellular calcium transient, and neurotransmitter release. PDE2A inhibition was also sufficient to reverse the abrogated BNP response. It was also observed that the stellate neurons of the prohypertensive rats express higher PDE2A levels as compared to the normotensive control. These data again underpin the importance of PDE2A upregulation in preventing the BNP-mediated inhibition of sympathetic transmission with subsequent maladaptive changes. Nevertheless, further experimental evidence is required to support whether the BNP-cGMP-PDE2A pathway is actually impaired in hypertensive and heart failure models.

More recently, Meier and colleagues [165] demonstrated the beneficial effect of CNP on β_1_- and β_2_-adrenoceptor signaling in rat hearts through cGMP-cAMP crosstalk, when PDE3 is inhibited by cGMP. The CNP-mediated interplay of the signaling pathways was unaffected both in healthy and failing hearts, while BNP was not able to regulate similar cAMP-mediated effects in any experimental group. This study analyzed mechanisms of cyclic nucleotide crosstalk, trying to explain the lack of long-term positive effects of natriuretic peptide in therapeutic schemes for heart failure.

In general, the use of family-selective PDE inhibitors and of genetic knock-down or knock-out models is another way to assess the contribution of individual PDE families in the compartmentalization of cAMP signaling pathways in cardiac myocytes [81,134,156,161,166,167]. It would be interesting to generate a tissue-specific PDE2 knock-out mouse line and explore the role of this particular phosphodiesterase in cardiovascular disease. This goal has remained unattainable due to perinatal lethality of global PDE2 knockout mice. The effect of PDE2 overexpression or upregulation e.g., by inflammation, remains to be clarified as to whether it counterbalances or further deteriorates cardiovascular disease in response to pathologic stimuli.

## 6. Conclusions

In conclusion, cAMP and cGMP signaling pathways as well as their crosstalk offer a high level of intracellular organization and constitute an interesting pharmacological topic in health and disease. The positive or negative cGMP-mediated regulation of cAMP response that occurs in intracellularly confined loci controlled by distinct PDE isoenzymes could potentially pave the way for novel pharmacological approaches in heart failure treatment. Despite the encouraging evidence, there is still a long way to go before we can fully decipher and understand the exact mechanisms by which these distinct molecular effectors maintain homeostasis and induce maladaptive changes in the heart.
